# Mutation in *Chek2* triggers von Hippel-Lindau hemangioblastoma growth

**DOI:** 10.1007/s00701-023-05825-x

**Published:** 2023-10-16

**Authors:** Jorge Cabrera-Montes, Daniel T. Aguirre, Jesús Viñas-López, Laura Lorente-Herraiz, Lucía Recio-Poveda, Virginia Albiñana, Julián Pérez-Pérez, Luisa M. Botella, Angel M. Cuesta

**Affiliations:** 1 Department of Neurosurgery, Sanitary Investigation Institute – Fundación Jiménez Diaz (IIS-FJD), Fundación Jiménez Díaz University Hospital, Madrid, Spain; 2 SECUGEN SL, CIB-CSIC, Madrid, Spain; 3 Department of Molecular Biomedicine, Center for Biological Research Margarita Salas, CIB-CSIC, Madrid, Spain; 4 Rare Diseases Networking Biomedical Research Centre (CIBERER), Unit, 707 Madrid, Spain; 5 Department of Biochemistry and Molecular Biology, Faculty of Pharmacy, Complutense University of Madrid, Madrid, Spain; 6 Health Research Institute of the Clínico San Carlos Hospital (IdISSC), Madrid, Spain

**Keywords:** von Hippel-Lindau disease (VHL), Rare cancer, CNS Hemangioblastomas, *Chek2*, ccRCC, Personalized medicine

## Abstract

**Purpose:**

Von Hippel-Lindau (VHL) is a rare inherited disease mainly characterized by the growth of tumours, predominantly hemangioblastomas (Hbs) in the CNS and retina, and renal carcinomas. The natural history of VHL disease is variable, differing in the age of onset and its penetrance, even among relatives. Unfortunately, sometimes VHL shows more severe than average: the onset starts in adolescence, and surgeries are required almost every year. In these cases, the factor that triggers the appearance and growth of Hbs usually remains unknown, although additional mutations are suspected.

**Methods:**

We present the case of a VHL patient whose first surgery was at 13 years of age. Then, along his next 8 years, he has undergone 5 surgeries for resection of 10 CNS Hbs. To clarify this severe VHL condition, DNA from a CNS Hb and white blood cells (WBC) was sequenced using next-generation sequencing technology.

**Results:**

Massive DNA sequencing of the WBC (germ line) revealed a pathogenic mutation in *CHEK2* and the complete loss of a *VHL* allele (both tumour suppressors). Moreover, in the tumour sample, several mutations, in *BRAF1* and *PTPN11* were found. Familiar segregation studies showed that *CHEK2* mutation was in the maternal lineage, while *VHL* was inherited by paternal lineage.

**Conclusions:**

Finally, clinical history correlated to the different genotypes in the family, concluding that the severity of these VHL manifestations are due to both, *VHL*-and-*CHEK2* mutations. This case report aims to notice the importance of deeper genetic analyses, in inherited rare diseases, to uncover non-expected mutations.

## Introduction

Von Hippel–Lindau (VHL) disease is an autosomal dominant tumoral syndrome caused by germline mutations of the *VHL* tumour suppressor gene (NC_000003.12) situated at chromosome 3 (3p25-p26) [8, 10].

With an incidence of 1 in 36,000 births [7], and an almost complete penetrance (>90%) by 65 years old [8], patients may develop hemangioblastomas (Hbs) at the central nervous system (CNS).

The natural history of VHL shows that the average onset is at 33 years old and that 80% of the patients will bear, at least, 1 CNS Hb in their lifetime [22, 24]. Furthermore, visceral lesions such as, clear-cell renal cell carcinoma (ccRCC), pheochromocytomas, pancreatic neuroendocrine tumours, and benign cyst adenomas of the adnexal organ, appear in those patients. The CNS tumours include Hbs of retina and the cranio-spinal axis (cerebellum, brain stem, and spinal cord) as well as endolymphatic sac tumours (ELSTs) [7, 18]. CcRCC and its metastasis constitute the most frequent cause of mortality associated to the VHL disease, while the development and progression of retinal Hbs and CNS Hbs are cause of a high morbidity and loss of quality of life, such as blindness due to retinal detachment and the need of surgeries to control the associated symptoms [9].

Without an effective treatment for VHL tumours, repeated surgeries become the first line treatment to tackle the disease [5]. Unfortunately, continued surgeries decrease the quality of life of the patient [6]. Inhibitors of VEGF/VEGFR and mTOR have been tested in clinical trials, showing a limited success [14]. More precisely, in advanced ccRCC, tyrosine kinase inhibitors such as Pazopanib have been used, but resistance appears as adaptation to the tumour environment [26]. Recently, belzutifan, a selective HIF-2α inhibitor for VHL RC, showed a limited response in a trial on 61 VHL patients suffering from early-stage non-metastatic ccRCCs [17, 26].

Clinical diagnosis of VHL is as follows: when there is a relative affected, the presence of a single CNS Hb, pheochromocytoma or ccRCC confirms the diagnosis; while in the absence of familiar history, the presence of 2 CNS Hbs, or 1 CNS Hb and other visceral tumour, is required. Obviously, the presence of a pathogenic mutation in the *VHL* gene by genetic testing is a definite criterion for the diagnosis [18].

Lonser et al. [21] and Dornbos et al. [11] described the natural history of VHL disease by means of a study including 225 VHL patients, which developed more than 2500 CNS Hbs, in a mean follow-up of 6.9 years, the observation ranging from 2.1 to 9.0 years. Most tumours (72 %) grew following a saltatory pattern (periods of quiescence followed by periods of growth). In a minor proportion, tumours progressed following exponential and linear patterns (22% and 6%, respectively) [11]. Finally, only 159 (6.3%) of all Hbs became symptomatic, requiring surgery during the follow up (6.9 years).

Zhang et al. aimed to elucidate the genotype–phenotype correlations and clinical outcomes in VHL patients with large deletions (LDs). They concluded that VHL patients with deletion in exon 2 had an earlier onset age of ccRCC and pancreatic lesion, but the risk of ccRCC was lower in VHL patients with LDs and a *BRK1* deletion. In addition, the group with earlier age of onset had poorer prognosis [19].

The histological origin of Hbs was unknown up to 2003 [18]. More recent investigations have shown that they may be originated from mesodermal derived hemangioblasts arrested during the embryonic development [15]. As normal embryologic hemangioblasts, Hb cells express markers of hemangioblasts. Among them, *TBXT* gene, called brachyury, a transcription factor within the T-box family of genes, VEGF receptor 2 (*Flk-1*), and stem cell leukaemia gene (*SCL*), which encodes a tissue-specific basic helix–loop–helix (bHLH) protein with a pivotal role in hemopoiesis and vasculogenesis [2]. The VHL tumour–derived stromal cells (hemangioblasts) can differentiate into hematopoietic and endothelial progenitors which may explain the anatomic distribution and variability of Hbs in VHL patients like the *SCL* axis of expression during embryogenesis [2].

Higher tumour burden has been recently associated to male sex and germline deletions. Patient with missense *VHL* mutations harbour less tumours than those with nonsense mutations and deletions [21]. The rate of Hb progression is significantly more rapid in symptomatic tumours and tumours with associated cysts [19, 21]. Patients younger than 20 years are significantly more likely to develop new tumours than those older than 40 years [21]. Consequently, surgical resection between 20 and 40 years could result in clinical stability since the risk of new tumour development decreases with age. Development of new tumours is also associated with the tumour burden at their presentation. A greater tumour burden may be indicator/consequence of other aggressive underlying pathological factors.

Knowledge and understanding of tumour pathophysiological features may help prognosis and improve the effectiveness of surgical and nonsurgical treatments and provide avenues for potential new therapies.

In this context, we studied the case of a VHL family with a member presenting a very early onset and a dramatic rate of Hb growth, requiring 8 surgeries in less than 10 years. This evolution of the disease contrasted with a more classical evolution in his relatives, bearing the same VHL familial mutation. To find a putative triggering factor, massive DNA sequencing in germline and in tumour cells was performed. An additional pathogenic mutation in a tumour suppressor gene was found, possibly becoming a modifying factor of the natural course of the disease.

With this work, we want to outline the importance of personalized analysis in medicine, especially in inherited rare diseases and oncology, to uncover non-expected mutations.

## Materials and methods

### Human samples

Blood samples and surgical surplus from members of a VHL family were processed in our lab. Informed consent from donors was obtained. The Ethical Committee of CSIC (Spanish National Research Council) approved all the procedures (references 075/2017 and 228/2020).

### DNA total extraction and sequencing

Total DNA was extracted from PBL (peripheral blood lymphocytes) and fresh tumour pieces using the QIAamp Mini Kit (Qiagen, Düsseldorf, Germany) and following the manufacturer indications. In the case of buccal swabs, DNA extraction was also made with QIAamp Mini kit (Qiagen) but with longer time of lysis in the first step. The panel used for the preparation of the library has been designed using SureSelectXT technology (Agilent Technologies, CA, USA), aimed at capturing the exons of the genes of clinical interest and the flanking splicing regions (5–20 bp). Sequencing of the library was performed on a next-generation mass sequencer, NovaSeq 6000 SystemTM (Illumina, CA; USA). The tumour sample was sequenced with a reading depth of 500×. The sequences obtained were aligned against the reference genome (GRCh38/hg38) and filtered according to specific quality criteria. Subsequently, they have been analysed for the identification of genetic variants included in exonic regions or splicing regions (at least 5 bp), including missense or nonsense mutations, synonymous mutations, indels, small insertions, or deletions found at a higher allele frequency (> 30% of germline reads and > 5% of tumour sample reads). Both processes were carried out using the DRAGENTM BioIT Platform software (Illumina, version 07.021.510.3.5.7). The identified variants were filtered and narrowed down to the study genes using the bcftools view tool (Developed by Li et al. version 1.15.1) [27].

Variant annotation was performed using the freely available online platform wANNOVAR (WGLAB, PA, USA), which compiles the main databases major databases such as ClinVar (with specific information on variants associated with a known genotype) and databases of population frequency data -dbSNP, gnomAD (Genome Aggregation Database), 1000 Genome Project, or NHLBI-ESP 6500 exons.

The pathogenicity of the variants was also estimated using CADD and the addition of selected prediction systems included in the dbNSFP database (SIFT, PolyPhen2, Mutation Taster, Mutation Assessor, LRT, FATHMM, and MetaSVM) for missense mutations. For mutations identified in splicing regions (including synonymous mutations), the effect on mRNA processing has been assessed using the SpliceAI [13], Splice Site Finder and Max-Ent-Scan prediction systems, included in the SPiCE algorithm. The conservation of nucleotide position has been evaluated according to the UCSC score ranges for the PhyloP tool.

Finally, the association of the identified variants with OMIM syndromes has been evaluated (date updated to 24 April 2022).

The nomenclature and classification of the variants is based on the guidelines of the Human Genome Variation Society (HGVS) (http://varnomen.hgvs.org/) and the American College of Medical Genetics and Genomics (ACMCG).

The analysis of CNVs (copy number variations) is a screening performed with the established parameters based on a set of control samples with the DRAGEN software (version 07.021.572.3.6.3). This algorithm allows the identification of non-recurrent CNVs associated with the patient’s phenotype following the quality criteria.

### Magnetic resonance imaging

MRIs were performed using a SIEMENS, Magneton Verio, 3 Teslas, 32 channel antenna, analysed by Numaris 4 Software (SIEMENS, Munich, Germany).

For acquisition of brain MRI images from the patients, a specific VHL protocol was used, including axial T1, axial T2, axial FLAIR, T2* (gradient echo), DW (diffusion-weighted), axial T1 SPIR (fat suppression, contrast-enhanced), and 3D T1 SPACE (iso, contrast-enhanced). For the posterior fossa study (endolymphatic sac tumours), thin sections of axial T1, axial FLAIR, and 3D T2 SPACE were obtained.

For acquisition of complete spinal cord MRI, a specific VHL protocol was used, including sagittal T1, sagittal T2 Dixon, axial T2, and sagittal and axial T1 SPIR (contrast-enhanced).

## Results

### Genetic analysis of VHL gene in a family

**Fig. 1:**
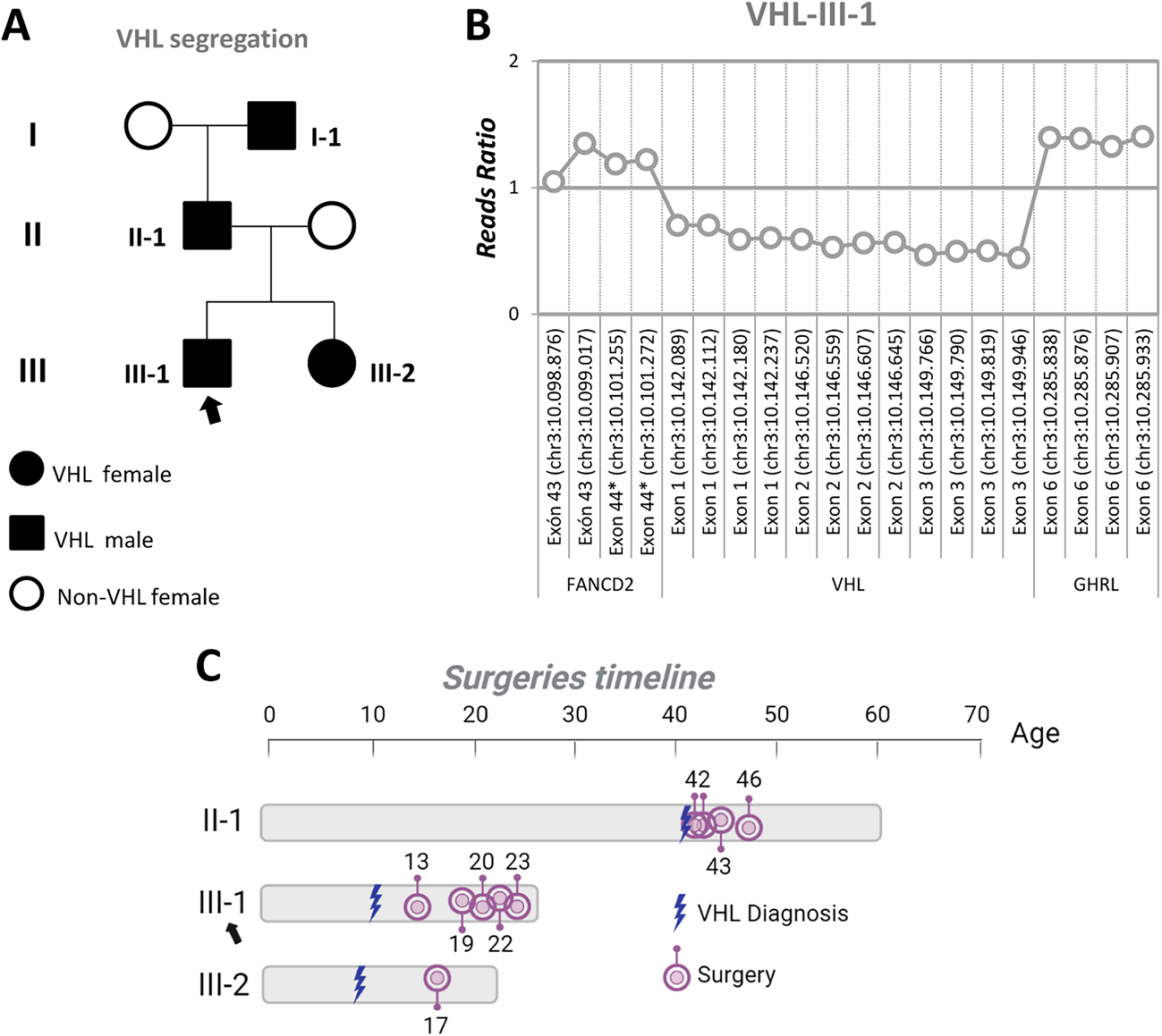
Familiar pedigree for *VHL* mutation. Reads ratio representation from the Integrative Genomics Viewer (IGV) of VHL-III-1. Timeline of surgeries of VHL patients of the pedigree

The *VHL* main case of study is a 23-year-old male, third generation with VHL disease and genetic diagnosis at 11 years of age. The family mutation consisted of a complete deletion of one *VHL* allele, being the patient hemizygous for the *VHL* gene in the germ line. The loss occurred on chromosome 3 at the 3p25.3 cytoband, in the genomic region chr3:10141778-10149995 (GRCh38). This mutation was inherited through the parental lineage and was also present in his sister. The VHL disease segregation among the members of the family is shown in Fig. 1A (pedigree).

The patient III-1, case of this study (Fig. 1A, black arrow) bears a complete gene deletion. In Fig. 1B, the interactive tool Integrative Genomics Viewer, (IGV) for the visual exploration of genomic data was used. He was primarily attended at other centre and then referred to our VHL unit at FJD, for follow-up and treatment of CNS tumours derived from VHL disease.

### Clinical manifestations of the VHL affected members

Table 1 shows the different surgeries undergone by the VHL-affected members of the family, including: age at the VHL disease onset, date of each surgery, and type of tumours resected from the father of the patient (II-1); the patient, main case of study (III-1); and his sister (III-2). In Fig. 1C, a timeline scheme of the surgeries is shown for better comparison and easier understanding of the clinical courses of these 3 patients.

**Table 1:** CNS surgeries in the three members of a VHL family

Patient	Relationship	Age	Surgery date	Type of tumour/number
II-1	Father	42	Nov. 2009	**Hemangioblastomas (2)** Cerebellar/hemisphere
			May 2010	**Hemangioblastomas (2)** Medulla oblongata/cervical spinal cord
			Dec. 2010	**Hemangioblastomas (4)** Cerebellar vermis/cerebellar hemisphere/medulla oblongata (obex + floor of IV ventricle)
			Jan. 2014 (2 different approaches in the same day)	**Hemangioblastomas (3)** 2 Medulla oblongata/1 cervico-medullary junction **Hemangioblastomas (1)** Cerebellar
III-1	Son	13	March 2013	**Hemangioblastomas (3)** 2 Medulla oblongata/1 cervical spinal cord
			Oct. 2018	**Hemangioblastoma (1)** Conus medullaris
			March 2020	**Hemangioblastomas (3)** 2 Medulla oblongata/1 cervical spinal cord
			Oct. 2021	**Hemangioblastoma (1)** Cervical spinal cord
			Oct. 2022	**Hemangioblastomas (2)** Intramedullary dorsal/spinal cord (D3+D5)
III-2	Sister	17	May 2018	**Hemangioblastomas (2)** Medulla oblongata

**Fig. 2:**
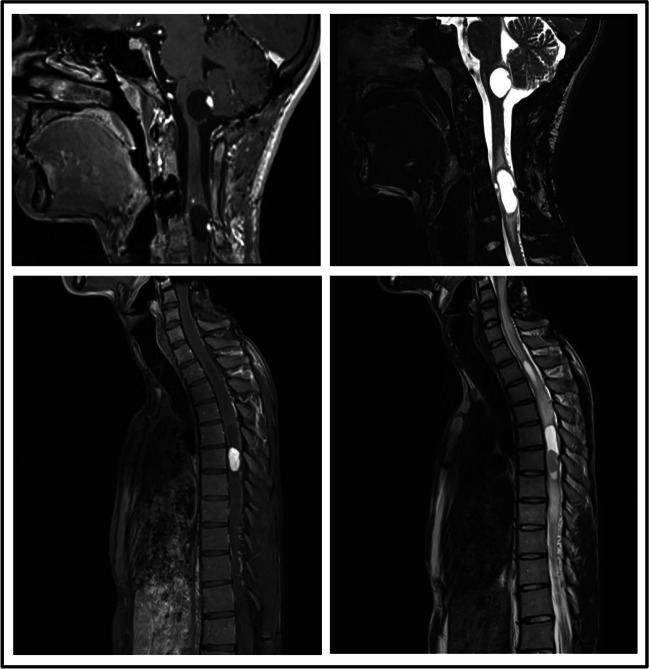
Contrast-enhanced T1 and T2 MRI sagittal cervical and dorsal images obtained from patient III-1, showing some of the hemangioblastomas with associated cyst that the patient presented

Figure 2 shows MRI images of different tumours present in the main case of study prior to surgeries.

### Patient I-1

The index case of VHL disease. He was clinically diagnosed late in age, and no clinical data from this patient are available.

### Patient II-1

A 55-year-old male VHL patient with onset of the disease at 42 years of age, genetically diagnosed with a complete allelic loss of *VHL*. This patient underwent his first surgery in other centre with removal of 2 hemispheric cerebellar Hbs in 2009, requiring a ventriculo-peritoneal shunt due to hydrocephalus. In 2010, he underwent a second surgery at FJD for removal of two Hbs located on the posterior medulla oblongata and upper cervical spinal cord. Ending 2010, additional surgery took place for removal of two other Hbs at the brain stem (nested in the obex and floor of IV ventricle), and two cerebellar Hbs (paravermal and right hemisphere). In 2014, a new surgical intervention was performed with removal of three tumour nodules, the largest in posterior medulla location and two smaller ones in a left medullary location. Then, under the same anaesthetic procedure, a second surgical intervention was performed with a left retro-sigmoid approach, for removal of a fourth tumour in the cerebellar hemisphere. Postoperative period followed without neurological deficits. Since 2014 until now, no further neurosurgeries have been needed.

### Patient III-2

A 21-year-old woman, genetically diagnosed with VHL disease at the age of 9, carrier of a pathogenic mutation inherited from her father (patient II-1), who was referred in 2014 for MRI evaluation at the VHL unit of the FJD at the age of 12, being asymptomatic at that moment. In 2017, MRI showed 2 Hbs in obex (medulla oblongata) and right para-medullary region, both of 5 mm, without associated cyst. A full spine MRI showed absence of Hbs in spinal cord. Conservative management was decided with programmed MRI follow-up after 1 year. In 2018, the patient started with episodes of hiccups, coughing, and choking, presenting enlargement of the lesion located in obex (8 mm of size), while maintaining unchanged the right 5mm HGB in para-medullary region. Surgical intervention was decided to avoid the progression of symptoms and a permanent neurological deficit. The patient underwent surgery, achieving the removal of the two medullary tumours, without complications. No further neurological surgeries have been necessary since then.

### Patient III-1

Son of patient II-1 and genetically diagnosed of VHL at 11 years old, asymptomatic and without tumours at the time of diagnosis. During his follow-up in 2012, an MRI showed for the first time the presence of a small left lateral medullary nodule, as well as a 7.5mm nodule in the posterior medulla oblongata. The latter grew quickly reaching 11mm in a year. Given the rapid growth and location of the lesion, he underwent a first surgery in 2013 to resect 2 medullary and 1 cervico-medullar Hbs. After surgery, the patient presented some transitory sequelae with slight loss of finger sensitivity at the right hand, and slight dysmetria in the left upper limb with complete recovery in consecutive revisions. Three months after the intervention, the patient presented a surgical wound infection and post-laminoplasty cervical kyphosis, requiring reoperation for cleaning, with anterior cervical arthrodesis C4–C5 and posterior instrumentation.

**Fig. 3:**
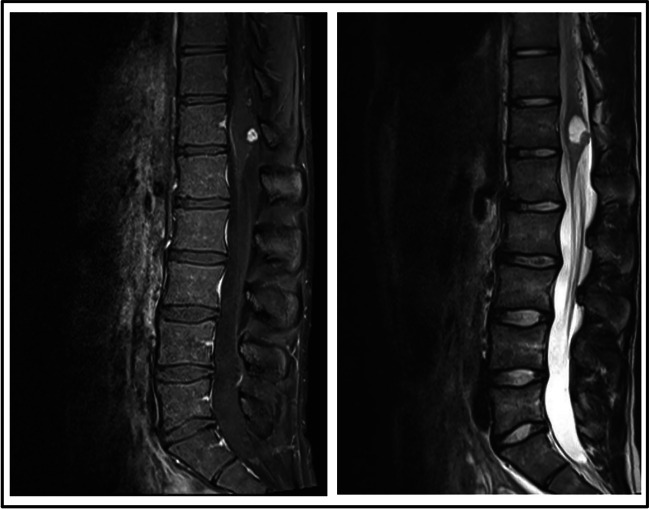
Contrast-enhanced T1 and T2 MRI sagittal images obtained from patient III-1, showing a hemangioblastoma with associated cyst located in the conus medullaris that required surgery

The patient remained stable for 18 months. Then, in 2015, a brain and spine MRI exam revealed the presence of a subcentimetric Hb on the right lateral medulla, without associated cyst; two more lesions were detected at C3–C4 and C6, without associated cysts; as well as a nodular Hb at the posterior conus medullaris, with associated edema and thickening of the cone. In August 2016, while the cervical and cone lesions remained stable, the lesion in the right margin of the medulla showed a millimetric growth. The lack of significant symptoms plus the millimetric growth, led to a conservative management for the next 2 years of follow-up. However, in 2018, an MRI of the whole spinal cord showed an increase of the conus medullaris lesion, with significant growth of the cyst and edema at that level and the surrounding spinal cord respectively (Fig. 3). These findings conditioned a new surgery with a complete resection of the tumour and an adequate post-surgical evolution. Nevertheless, 6 months later, in 2019, the patient presented paraesthesia on the left upper limb with radiological stability and consequently a conservative management. In January 2020, the patient presented a worsening of the neurological symptoms in the extremities. Imaging tests showed an increase in the size of the medullary lesion, as well as oedema surrounding the associated cyst. Likewise, an increase in the size of the cyst over the C4–C5 lesion was apparent, with medullary bulging and perilesional edema. Given these symptoms, in March 2020, surgery was performed on the medullary and cervical lesions, resecting a total of 3 Hbs (1 medullary and 2 cervical), with no complications. Postoperatively, the patient presented alterations in proprioceptive sensitivity and coordination in the right upper limb, which improved during hospitalization.

The follow up of the patient showed progressive improvement of the sensitive deficit in the right hand, but a persistent decrease in manual ability. The brain lesions remained stable, but significant growth of the cystic area associated with the C5 anterior Hb was apparent in MRI of the cervical spine. Conservative management was decided on this occasion. However, the evolution was to continuous cervicalgia with pain radiating to both trapezia. Given the growth of the cervical lesion cyst, the patient’s symptoms, and the surgical accessibility by posterior approach, a new intervention was decided. In October 2021, surgery was performed, and the lesion was resected without associated complications. In October 2022, the patient presented a rapid clinical worsening with uncontrollable pain at the level of the left costal grid. An MRI of the dorsal spine was performed showing significant growth of a previously known dorsal intramedullary Hb, with a large cystic cavity and associated myelopathy that had remained stable until that moment (Fig. 2). Given the radiological and clinical myelopathy, a new surgery was practiced in December 2022, achieving the removal of the lesion at D3 level, without complications and with adequate post-surgical evolution.

In the last MRI evaluation, in February 2023, a growth of the right cerebellar lesion was evident, with probable future surgical management, given the progressive growth of the lesion and the patient’s coordination alterations.

### Genetic analysis from tumoral and blood DNA by NGS

Looking at the clinical symptoms of this family, (Table 1), especially at the onset age and the number of surgeries/tumours, the case of patient III-1 is striking by the early and severe VHL presentation.

In certain cases of VHL disease, the fast growth of Hbs may occur due to the accumulation of the already present loss of function (LOF) of the *VHL* gene with somatic mutation(s) in other tumour-related gene(s). To investigate the possibility of additional mutations in these genes, a massive sequencing analysis of total DNA from a piece of Hb coming from the last surgery (Table 1) in cerebellum of patient III.1, was performed. The massive sequencing, with a high level of readings, yielded three different mutated genes present at this tumour, in addition to the already known mutation in the inherited *VHL* gene.

The variant c.1259+1G>C (NM_007194.4, rs121908707) was detected in heterozygosis in the *CHEK2* gene, affecting the first nucleotide of intron 10, interfering with the splice site leading to skipping of exon 10, loss of the reading frame and a premature termination of the protein sequence (p.I336Pfs*2). In silico CADD (combined annotation dependent depletion) indicated a high predictor value of 34, pointing to high probability of pathogenicity.

**Fig. 4:**
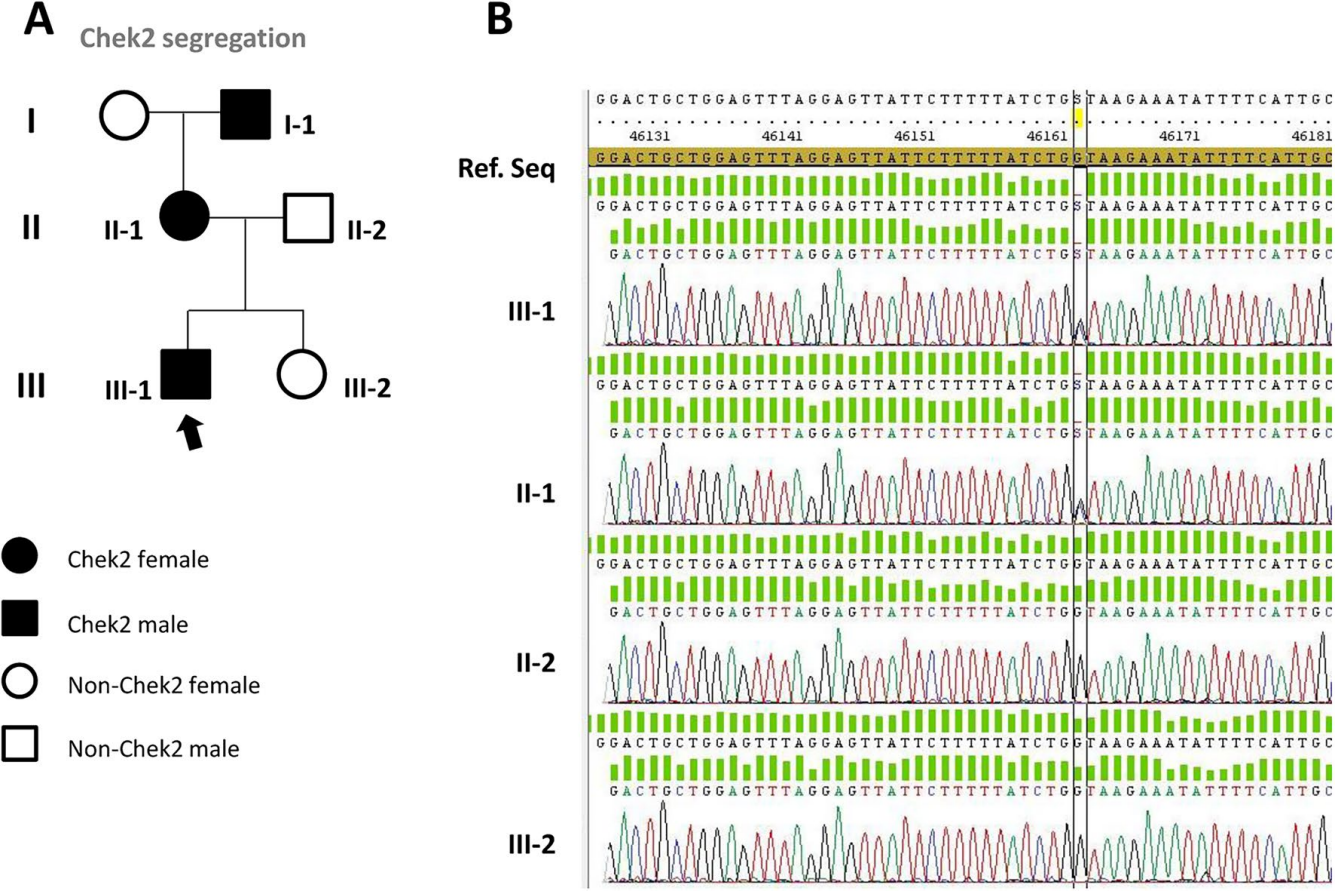
Familiar pedigree and the Sanger sequencing of *CHEK2* DNA. As seen in the DNA sequencing chromatogram, *CHEK2* mutation was found in the mother but absent in the father and the sister, both VHL patients

Analysis of the DNA from peripheral blood indicated the presence of the same mutation in the germ line, as shown in Fig. 4B (III.1).

To determine whether the mutation was inherited from the parents or was a de novo mutation in patient III.1, segregation analysis of *CHEK2* mutation in the family was studied. Figure 4A shows the familiar pedigree and the Sanger sequencing of *CHEK2* DNA from saliva in both parents and his sister. As seen in the DNA sequencing chromatogram, *CHEK2* mutation was found in the mother but absent in the father and the sister, both VHL patients. Of note, his mother had been diagnosed of an in situ breast carcinoma, resected 5 years ago, and his maternal grandfather was diagnosed of prostate cancer. In both cases the presence of tumours correlated to the finding of the *CHEK2* mutation.

In addition to *CHEK2* mutation found in the tumour and inherited through the germ line, 2 more somatic mutations were detected only in the tumour sample.

The analysis of CNVs in the tumour sample showed the presence of a deletion in exon 1 of *BRAF*. The loss occurs on chromosome 7, at cytoband 7q34, in the genomic region chr7:140924459-140924753 (GRCh38). The *BRAF* proto-oncogene encodes a protein of the RAF family, composed of serine/threonine kinases that mediate cellular responses to grow signals by activating the mitogen-activated protein kinase pathway (MAPK). Additionally, the analysis also showed the presence of a deletion in exon 1 of *PTPN11,* a protein member of the protein tyrosine phosphatase (PTP) family. The loss occurs on chromosome 12, at cytoband 12q24.13, in the genomic region chr12:112419022-112419200 (GRCh38). The potential consequences of these mutations will be explained in the discussion section.

## Discussion

Rare diseases are characterized by the low number of patients suffering from each individual disease, but also by the scarce knowledge at clinical and research level, due to their rarity. In addition to their condition, it must be considered that these patients are not free from other diseases or mutations that affect general population. Particularly, in the case of VHL disease, we have published the case of a 76-year-old woman in which a mutation in *CLN5* (ceroid lipofuscinosis, neuronal, 5) offers a protective effect, preventing VHL-related tumour development [4]. Here, we introduce the other side of the coin, a mutation that could dramatically affect Hb CNS tumours raising and development.

In RDs, there are two main goals: an early diagnosis and the natural history knowledge of each disease, which contribute to a better clinical management and to find therapies improving quality of life. Knowledge of the natural history of the disease is a key point for its clinical management. For this purpose, the medical record documents from patients and review studies from them, would allow to know the expected evolution of a rare pathology.

In the case of VHL disease, makes mandatory a follow up of patients by experienced clinicians.

As mentioned in the Introduction section, surgery arises as the only way to resolve the symptoms of VHL disease. Since patients have multiple asymptomatic/symptomatic tumours in the context of the disease, surgeons must follow a conservative approach, and only remove those leading to life-threatening symptoms, deciding the proper timing in each case.

To describe and publish the natural history of CNS Hb development in VHL patients is extremely useful. Lonser et al. indicated that in 70–80% of cases, the common pattern is an evolution of growth periods followed by quiescence phases in tumour development [21].

Several clinical trials have been carried out with drugs, most of them used in cancer chemotherapy to stop and space the need of surgeries, extending the periods of tumour quiescence (angiogenesis inhibitors, HIF-2 dimerization inhibition, β-blockers, etc.). However, no clearly positive results are available so far [14].

The present work describes the particularly severe case of a VHL patient, with a very early disease onset, at the age of 11 years, and with the need of a first surgery at the age of 13 years old. Since then, a conservative surgical basis has been applied, removing only those tumours with severe clinical impact on the patient. A total of 8 neurosurgeries have been necessary, and he is currently 23 years old. The patient inherited paternally the complete deletion of a *VHL* allele, but both, father and grandfather had the disease with a much later onset, presenting their first symptoms after the age of 40. Regarding the father, his first surgery of the CNS was at the age of 42 and, as shown in Table 1 and Fig. 1, in a period of 4 years he underwent several surgeries. However, notably since 2014, he has remained without the need for new interventions.

It could be hypothesized that in patient III-1 (Fig. 1), there is a phenomenon of gene anticipation as published in VHL [25]. However, the present work has another explanation. In fact, the present work is a paradigmatic case of how useful a personalized medicine approach is. By massive genetic sequencing analysis of DNAs from both, tumour, and germ line (peripheral blood), we have discovered the presence of two germline mutations in the patient. He inherited an allelic loss of *VHL* from the paternal side and a pathogenic mutation in the tumour suppressor gene *CHEK2* from the maternal side.

*CHEK2* encodes the Chek2 protein kinase activated in response to DNA damage, being involved in cell cycle arrest. Mutations in *CHEK2* are responsible for an increased predisposition to breast, prostate, colon, stomach, and brain cancer [20]. In relation to VHL, it has been shown that *CHEK2* binds to the β-domain of pVHL and phosphorylates it upon DNA damage. Therefore, this modification enhances pVHL-mediated transactivation of p53, recruiting p300 and Tip60 to the chromatin of p53 target gene [23]. Moreover, CHEK2 functions as a DNA damage checkpoint kinase by phosphorylating p53 [16]. Bell et al. [20] described heterozygous germline mutations in the *CHEK2* gene in patients with Li-Fraumeni syndrome, suggesting that *CHEK2* is a tumour suppressor gene whose loss of function confers predisposition to develop sarcoma, breast cancer, and brain tumours. The *CHEK2* variant found in our case had been previously identified and described in patients with non-Hodgking’s lymphoma by Havranek et al. [1].

Upon finding the *CHEK2* mutation, it was hypothesized that this mutation would not be present in the rest of the family siblings carrying the *VHL* mutation. Thus, the segregation of *CHEK2* in the family germline was carried out. As expected, it was observed that the mutation came from the maternal line (pedigree II-2). It is worth mentioning that the mother underwent a breast cancer surgery several years ago. In the family, the only case where pathogenic mutations in both genes, *VHL* and *CHEK2*, are concomitant was in patient III-1, showing an unusual and very severe clinical history of VHL disease. We, therefore, conclude that the mutation in *CHEK2* is the factor triggering the rapid growth of CNS Hbs in this patient. Furthermore, at the time or writing this manuscript, Zhang et al. found two VHL patients with LDs also carrying *CHEK2* and *FLCN* germline mutations, respectively [19].

Moreover, in other pathologies such as familial cavernomatosis, it has recently been described that somatic mutations in *PIK3CA* trigger the growth and bleeding of cavernomas that lead to surgeries [3]. Considering the evolution of the patient and the differences with respect to first-degree relatives (bearing a VHL mutation but lacking the *CHECK2* mutation), is very likely that the combination of mutations in two different tumour suppressor genes is responsible for the more aggressive behaviour of the disease in patient III-1. Following the clinical-genetic finding, the family has been referred to familiar oncology, to study the possible benefit derived from *CHEK2* mutation–targeted therapy, which could stop the continuous triggering of tumour growth.

In addition, a complete genetic analysis of the tumour revealed not only the presence of *CHEK2* and *VHL* mutations, but also the presence of deletions in exon 1 of *BRAF-1* and *PTEN11*, genes involved in tumour development by LOF. It is worthy to remark that these mutations were not present in the germline.

In the case of *BRAF-1*, a key intermediate in the RAS pathway and in the transmission of signals that regulate cell proliferation, differentiation, and survival, exon 1 corresponds to the N-terminal regulatory domain that precedes the Ras-binding domain of BRAF. In the work of Martínez-Fiesco et al. [12], they suggest that this region may represent an additional level of regulation in terms of RAS-BRAF interaction. Moreover, Terrell et al. [23] reported a high affinity of BRAF for KRAS; however, they observed that BRAF proteins lacking the N-terminal domain had an increased affinity for other RAS family proteins different from KRAS, such as HRAS and NRAS.

PTPN11, as PTP protein, regulates a variety of cellular processes, including cell growth, differentiation, the mitotic cycle, and oncogenic transformation. Mutations in *PTPN11* lead to the activation of the RAS-MAPK pathway. Exon 1 is included within the SH_2_ N-terminal domain involved in the switching of the protein between its inactive and active conformations. Therefore, alterations in this domain can cause a significant shift in the balance, favouring the active conformation and, therefore its malignancy.

As mentioned above, another interaction in the germline was described by our group recently [4]. Fortunately, the opposite effect to the present case was produced since the tumour development due to a *VHL* mutation was counteracted by a germ line mutation in heterozygous condition at the *CLN5* gene [4]. These findings of gene interactions between *VHL* and other genes in the germline underlines the need to study the whole exome in those cases in which the disease does not follow a natural course. Therefore, completing the two main points above indicated (diagnosis and natural history of the disease), massive germline and tumour NGS study should be done with all VHL patients, starting from their first surgery.

Advances in genetics and molecular biology techniques make it advisable to apply personalized medicine to monitor and treat each patient according to their genetics and associated symptoms.

Thus, by showing this case, we propose deeper genetic studies, searching for the origin of tumoral abnormal growth outside the natural history. In many cases, if we were able to find the cause, maybe the quiescence of the disease could be reached, by treating patients according to the affected gene. The study of genetic factors that can trigger the development of tumours through massive sequencing can help to modify the natural evolution of genetic tumoral diseases like VHL, in a personalized way. On the other hand, we cannot rule out that there may be also environmental or epigenetic causes triggering Hbs’ growth, and that should also be considered.

## Conclusions

The present work shows the importance of personalized medicine in, the case of a VHL patient bearing a second inherited mutation, which we propose as the trigger for the growth of his CNS hemangioblastomas. *VHL* is a tumour suppressor gene, and we postulate that the cause of the abnormal severity in the course of his disease, is an additional mutation in *CHEK2*, another tumour suppressor gene. Consequently, the onset of the disease is sped up to 11 years old and, along the following 12 years, he underwent 5 surgeries (10 hemangioblastomas), while his VHL mutation-bearing relatives (father and sister) had a much later onset and underwent 4 and 1 surgeries, respectively, in their life.

Thus, our objective is to provide an explanation why some VHL patients have an accelerated growth in their hemangioblastomas and to highlight the importance of exhaustive genetic analyses to uncover mutations in additional genes that can modulate the development of the disease and establish therapeutic targets.

## Data Availability

Not applicable.

## Abbreviations and acronyms

ACMCG: American College of Medical Genetics and Genomics
bHLH: Basic helix–loop–helix
CADD: Combined annotation dependent depletion
ccRCC: Clear cell renal cell carcinoma
CLN5: Ceroid lipofuscinosis neuronal
CNS: Central nervous system
CNVs: Copy number variations
DW: Diffusion-weighted
ELST: Endolymphatic sac tumour
Hbs: Hemangioblastomas
HGVS: Human Genome Variation Society
HIF: Hypoxia inducible factor
IGV: Integrative Genomics Viewer
LOF: Loss of function
MRI: Magnetic resonance imaging
NGS: Next generation sequencing
PBL: Peripheral blood lymphocytes
PTP: Protein tyrosine phosphatase
RBD: Ras-binding domain
RC: Renal carcinoma
RD: Rare disease
SCL: Stem cell leukaemia
SNPs: Single nucleotide polymorphisms
VEGF: Vascular endothelial growth factor
VEGFR: Vascular endothelial growth factor receptor
VHL: von Hippel-Lindau
WBC: White blood cells
